# Mechanistic Targets and Nutritionally Relevant Intervention Strategies to Break Obesity–Breast Cancer Links

**DOI:** 10.3389/fendo.2021.632284

**Published:** 2021-03-17

**Authors:** Ximena M. Bustamante-Marin, Jenna L. Merlino, Emily Devericks, Meredith S. Carson, Stephen D. Hursting, Delisha A. Stewart

**Affiliations:** ^1^ Department of Nutrition, University of North Carolina, Chapel Hill, NC, United States; ^2^ Nutrition Research Institute, University of North Carolina, Kannapolis, NC, United States

**Keywords:** obesity, breast cancer, inflammation, metabolism, immunosuppression, hormone signaling, nutrition

## Abstract

The worldwide prevalence of overweight and obesity has tripled since 1975. In the United States, the percentage of adults who are obese exceeds 42.5%. Individuals with obesity often display multiple metabolic perturbations, such as insulin resistance and persistent inflammation, which can suppress the immune system. These alterations in homeostatic mechanisms underlie the clinical parameters of metabolic syndrome, an established risk factor for many cancers, including breast cancer. Within the growth-promoting, proinflammatory milieu of the obese state, crosstalk between adipocytes, immune cells and breast epithelial cells occurs *via* obesity-associated hormones, angiogenic factors, cytokines, and other mediators that can enhance breast cancer risk and/or progression. This review synthesizes evidence on the biological mechanisms underlying obesity-breast cancer links, with emphasis on emerging mechanism-based interventions in the context of nutrition, using modifiable elements of diet alone or paired with physical activity, to reduce the burden of obesity on breast cancer.

## Introduction

Obesity is a state of increased adiposity defined by a body mass index (BMI) ≥ 30 kg/m^2^ (1). Current global estimates suggest that 1.97 billion adults are overweight (BMI =25.0-29.9 kg/m^2^) and over 650 million are obese (2). By 2030, it is estimated 57.8% of the global adult population will be overweight or obese if current trends continue (3, 4). The impacts of obesity on human physiology include dysregulation of insulin, bioavailable insulin-like growth factor (IGF)-1, adipokines (e. g. leptin and adiponectin), inflammatory factors (e. g. cytokines), and vascular integrity-related factors (e. g. vascular endothelial growth factor (VEGF)) (3, 4). As a result, multiple diseases are associated with obesity, including hypertension, dyslipidemia, type 2 diabetes, coronary heart disease, and several cancers (5).

Breast cancer (BC) is the most common cancer in women worldwide (6). As a heterogeneous disease, BC subtypes have been extensively described elsewhere (7). Briefly, the intrinsic subtypes are classified by hormone receptor positive/human epidermal growth factor receptor 2 negative (HR^+^/HER2^-^, Luminal A), HR^+^/HER2^+^ (Luminal B), HR^-^/HER2^+^ (HER2-enriched), HR^-^/HER2^-^ (basal-like or triple negative breast cancer, TNBC), and claudin-low (TNBC-metaplastic) (8, 9). Worldwide, there were over 2 million new BC cases in 2018 (6); and global epidemiological patterns show the importance of cultural and lifestyle factors, with only 5-10% of BCs being inherited (10). An estimated ~20-50% of BC can be attributed to modifiable risk factors, including physical inactivity and nutritional choices that result in obesity (11).

Obesity-driven BC risk is associated with multiple factors including menopausal status (12). Further, the risk of developing postmenopausal BC is exacerbated by obesity (13). These women have worse disease-free and overall survival despite appropriate therapeutic approaches. Results from a meta-analysis of 34 cohort studies with over 2.5 million women found that with each 5 kg/mg^2^ increase in BMI, there is a 12% increase in risk of postmenopausal BC (14). The risk is further dependent on other factors including, BC subtype (15, 16), race/ethnicity (16–18), estrogen and progestin use (18) and hormone receptor status (19). Obese BC patients also experience complications during surgery, radiation, and chemotherapy, and are at increased risk for local recurrence. Additionally, there is a greater probability for increased tumor size, metastatic rates, resistance to endocrine therapy, and advanced disease stage at diagnosis (19, 20). While obesity is known to increase postmenopausal, HR^+^ BC risk (13), more recent studies assessing central adiposity revealed that high abdominal obesity increases risk for ER- and TNBC in premenopausal women (13, 16, 21, 22). Similarly, in preclinical animal model studies, mammary tumor development and progression of HR^+^, basal-like and claudin-low subtypes is exacerbated by obesogenic environments (1, 23). In contrast, only minimal clinical and pre-clinical data supports that the Luminal B and HER2 subtypes, are enhanced by obesity (1, 24).

Here we focus on nutritional and physical activity-based interventions shown to ameliorate obesity-associated enhancements of growth signaling, inflammation, angiogenesis, and metastatic processes in BC. We also discuss gaps and potential uses of these strategies to mitigate obesity pro-BC effects.

## The Role of Growth Factor and Hormone Signaling in Obesity-Breast Cancer Links

Overweight and obese patients have increased risk of developing hormone and growth factor perturbations resulting in insulin resistance, increased production of estrogen, enhanced insulin-like growth factor (IGF)-1 bioavailability and a decreased adiponectin/leptin ratio. These can all increase BC incidence, tumor development, progression, and worsen clinical outcomes.

### Insulin and Insulin-Like Growth Factor-1

**Figure 1:**
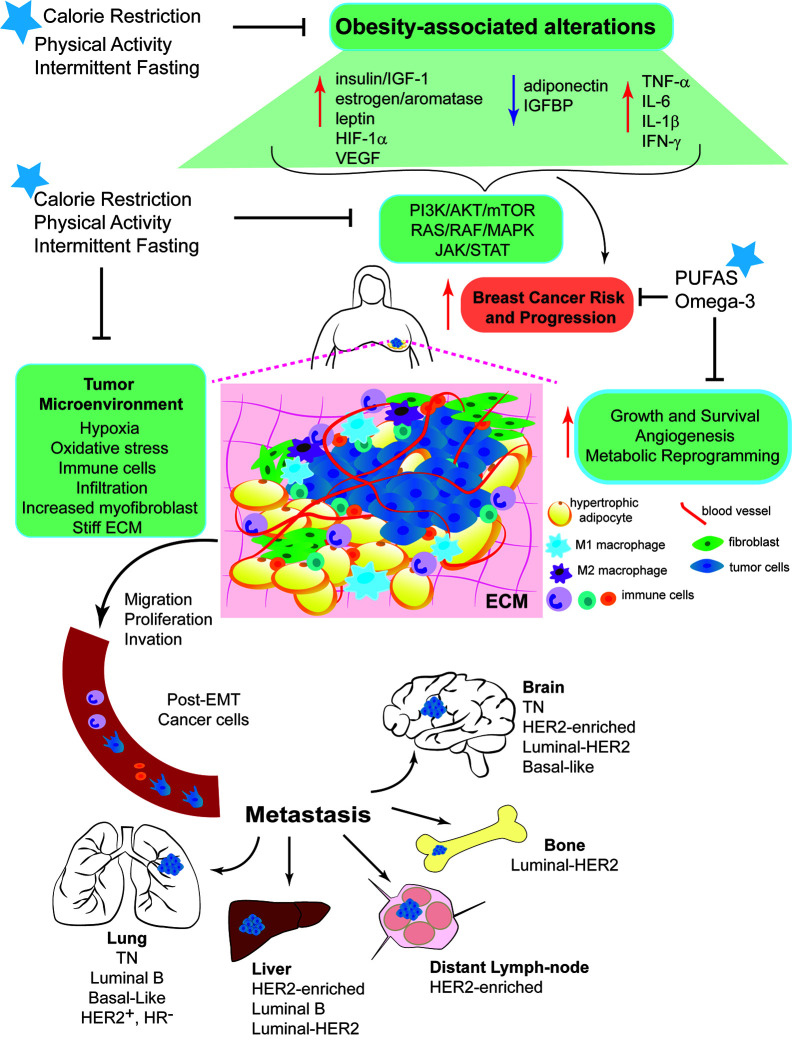
Mechanisms linking obesity with breast cancer development and intervention strategies to break Obesity-Breast Cancer Links. Increased energy intake and low physical activity results in obesity. Excess adiposity causes systemic changes, such as increased circulating levels of insulin/IGF-1, aromatase activity, estrogen production, and leptin:adiponectin ratio. Local changes due to the hyperplasia and hypertrophy of adipocytes, leads to a pro-inflammatory response promoting the secretion of cytokines and inflammatory molecules. These systemic and local changes activate key signaling pathways (PI3K/AKT/mTOR, RAS/RAF/MAPK, and JAK/STAT). The complex interplay among all of these alterations generates a microenvironment favorable for breast epithelial cell transformation and increase breast cancer risk and progression. Dysfunctional adipocytes, distant and present within the tumor microenvironment, produce high levels of leptin that contributes to chronic inflammation and BC progression. Obesity promotes cell proliferation, migration and invasion, epithelial-mesenchymal transition (EMT), angiogenesis and recruitment of immune cells. Obesity increases myofibroblast content, which stiffens the extracellular matrix (ECM) and enhances cancer cell growth. All these effects stimulate the entry of invasive cells into the circulation and the subsequent metastatic colonization of distant organs, such as bone, lung, liver and brain. Nutritional interventions, such as calorie restricted diets with balanced protein content and intermittent fasting can break the obesity-BC links, the benefits of the dietary interventions can be further improved by increasing daily physical activity.

Most obese patients have high levels of insulin, increased bioavailable IGF-1, low levels of IGF binding proteins (IGFBPs), and higher steady state levels of mTOR activation (25, 26) (**Figure 1**). Insulin, produced by pancreatic beta cells and released in response to elevated blood glucose, predominantly mediates metabolic activity whereas IGF-1, primarily produced by the liver, controls long-term action to determine cellular fates. IGF-1 bioavailability is regulated by IGF binding proteins (IGFBPs) 1-6, which regulate IGF-1 binding to the IGF-1 receptor (IGF-1R) and cross-reactivity with the insulin receptor (IR) (27). The activation of IR and IGF-1R lead to downstream phosphorylation cascades that promote the PI3K/AKT/mTOR and RAS/RAF/mitogen activated protein kinase (MAPK) pathways. These signaling pathways are associated with cancer development and progression (1, 28, 29). The connection between insulin and BC risk has been shown in several meta-analyses (30, 31), suggesting that hyperinsulinemia and elevated basal insulin levels increase BC risk and are a negative predictor of BC prognosis (31–33).

### Estrogen and Aromatase

The production of estrogen in the adipose tissue of obese patients, secondary to increased aromatase activity, has been found to be a key driver of BC (21, 34). The rapid expansion of adipose tissue observed during weight gain in obese women causes a rise in pre-adipocytes expressing aromatase (35). The tumor microenvironment, rich with adipocytes, is also a source of estrogen production and aromatase expression in BC cases, and can contribute up to 10-fold increased levels of estrogen in breast tumors compared to levels in surrounding tissue (3). In ER^+^ BC, increased ERα promotes cell proliferation (36), and obesity is positively associated with ERα positive tumors (37).

### Leptin, Adiponectin, and Their Ratio

In the obese state, the dysfunctional adipose tissue overproduces the hormone leptin, causing leptin resistance (38, 39) and reducing the adiponectin/leptin ratio (40) (**Figure 1**), which is negatively associated with BMI (41, 42). High levels of leptin through its receptor, Lepr, activate the Janus kinase and signal transducer activator of transcription (JAK/STAT) pathway that is often dysregulated in cancer (43). Hyperleptinemia also stimulates mitogenesis, angiogenesis, and the secretion of proinflammatory cytokines such as interleukin (IL)-6, tumor necrosis factor (TNF)-α, IL-2 and interferon (IFN)-γ (44). High leptin levels and reduced adiponectin/leptin ratio were associated with an increased risk of postmenopausal BC in a multiethnic case-control study (45, 46). Similar results have been documented in preclinical models of BC (47).

### Interventions to Break Growth Factor and Hormone-Signaling-Associated Obesity–Breast Cancer Links

Many nutritional and lifestyle habits can modify BC risk. Physical activity, for example, has many benefits including promotion of weight loss, reduction of hormone levels (48, 49), regulation of insulin and IGF-1 bioavailability, and normalization of leptin/adiponectin ratio (50, 51). According to the National Cancer Institute, exercising for four or more hours per week decreases BC risk (52). This is supported by epidemiological studies that observed inverse relationships between physical activity and risks of BC (53, 54). Interventions designed to reduce calorie intake, such as, calorie-restricted diets and intermittent fasting, can reverse the high levels of insulin and IGF-1 (55–57) (**Figure 1**). However, there is no consensus regarding the impact of diet and exercise on IGF-1 and IGFPB. Although there are limited clinical studies in the field, a recent study in young obese females showed that 4-weeks of aerobic exercise (6 days/week, two hours twice a day) combined with a diet intervention (daily energy intake of 1,400 or 1,600 calories) reduced the serum levels of IGFBP-3 while increasing the activity of IGF-1; however the intervention did not affect the total serum levels of IGF-1 (58). A different study showed that 5 weeks of diet combined with 45 minutes of moderated-to-intensive exercise has no impact on IGF-1 and IGFBP levels in overweight or obese women, but the molar ratio IGF-1/IGFBP3 was significantly increased by the intervention (59). Better reduction of total IGF-1 serum levels might be achieved with longer intervention times. Low circulating levels of IGF-1 were reached after 16 weeks of strength training (60). High levels of IGF-1 could be lowered temporarily by bariatric surgery (55); however, for long term reduction of IGF-1 level, a nutritional intervention is necessary to maximize the effects of the surgery on IGF-1 levels (55, 61). Dietary interventions (55–57), in particular, a 2008 study by Fontana et al. demonstrated total caloric restriction (CR) and targeted protein reduction was needed to lower circulating serum IGF-1, albeit in a small study of six participants (56); however a recent meta-analysis of six clinical trials corroborated these findings in part, showing an increase in circulating IGF-1 in response to increased protein intake (57). Studies in mice have also shown that low circulating IGF-1 correlates with reduced mammary tumor volume (1, 62). Thus, further investigation is warranted on the role of CR and macronutrient ratio modification methods to determine whether the BC associated to obesity with IGF dysfunction can be disrupted by these strategies.

Regarding dietary components, BC researchers have long investigated the impact of nutritional strategies to decrease the risk of BC and improve treatment outcomes. For example, plant-based diet interventions, rich in fiber, antioxidants, and phytochemicals could reduce BC incidence. In recent years intermittent fasting has emerged as a strategy to reduce adipose tissue and improve insulin levels, which can also lower estrogen levels and slow the growth of breast tumors. It was shown that short-term fasting 24 hours before and after chemotherapy can reduce the cytotoxicity of neoadjuvant docetaxel/doxorubicin/cyclophosphamide treatment in HER2^-^ BC (63, 64). Intermittent fasting impacts multiple cancer related pathways (**Figure 1**), including reducing IGF-1 and increasing IGF1BP by negatively regulating growth hormone-mediated IGF-1 mRNA production (65, 66). Fasting lowers blood glucose and circulating insulin levels, this could result in suppression of PI3K/Akt and reduction of mTOR activity. Studies using mouse models have shown that mTOR inhibitors block the tumor-enhancing effects of obesity (67), indicating that mTOR inhibitors in combination with intermittent fasting could represent a potential strategy for breaking the obesity-BC link. Nutritional-dependent mitigation strategies to facilitate mTOR repression, could include the incorporation of cardamonin found in cardamom spice and other plants and flavonoids in the diet (68, 69).

## Mediators of Inflammation and Immunosuppression in Obesity–Breast Cancer Links

Chronic inflammation is an established hallmark of cancer (70–72), and represents a highly relevant mechanistic target for nutrition and cancer research (**Figure 1**). Stromal breast tissue communicates with tumor cells in the microenvironment to usurp homeostatic inflammatory and resolution mechanisms, increase genomic instability, and recruit immune cells, further propagating inflammatory signals and increasing cancer cell survival. Failure to resolve inflammation often occurs with obesity, and the mechanisms underlying this are under intense investigation (73). Well described inflammatory mediators include transcription factors (i.e., NF-κB), soluble signaling molecules (i.e., cytokines, chemokines, growth factors, and specialized pro-resolving mediators) and their receptors, and immune cell populations (e.g., tumor-associated macrophages and T-cells) (74–78).

### Cytokines and Pro-Inflammatory Mechanisms

Cytokines are small proteins that coordinate immune responses to assist with re-establishing homeostasis following insult or injury (79). Many cytokines have pleiotropic activity making interpretation of their expression patterns and downstream consequences difficult in the disease context (80, 81). However, several are useful biomarkers of systemic dysfunction when unchecked inflammation does not resolve, including angiogenin, IL-1β, IL-6, IL-17, osteopontin, osteoprotegerin, RANTES, TNF-α and TGF-β, linking persistent inflammatory mechanisms to arthritis, type 2 diabetes, obesity and cancer (82–85). NF-κB is a master regulator coordinating genetic, metabolic and inflammatory instabilities in many cancers, primarily by increasing cytokine levels and signaling, and these effects are typically more dysregulated in obese patients (86). Pro-inflammatory signaling can promote BC proliferation, angiogenesis, invasion, metastasis repress tumoricidal host immunosurveillance programs and decrease chemotherapeutic treatment response (87–91) (**Figure 1**).

Under obesogenic conditions, the crosstalk between dysregulated adipose tissue, inflammatory mediators and tumor cells can have adverse impacts on BC outcomes (92). Autocrine and paracrine signaling mechanisms drive anti- and pro-inflammatory signals within the tumor microenvironment, linking inflammation to tumor aggressiveness, disease progression and chemoresistance programs (92, 93). This communication, which also includes other stromal components (i.e., fibroblasts and tumor-adjacent normal tissue), creates a milieu promoting genetic instability that enhances every aspect of BC progression from increasing proliferation, reducing apoptosis, and facilitating angiogenesis, migration, and ultimately metastasis (94). In obese women, systemic abnormalities can also include comorbidities like nonalcoholic fatty liver disease, shown to interfere with expression of several cytochrome P450 genes involved in drug metabolism, contributing to obesity-associated reductions chemotherapeutic efficacy (95, 96).

### Obesity, Breast Cancer, and Immunosuppression

Breast tumors are usually infiltrated by multiple immune cell populations, most notably macrophages, referred to as tumor-associated macrophages (TAMs), as well as neutrophils and T-cells, that can reduce treatment efficacy through immunosuppressive mechanisms (76, 97). Grimm and colleagues reviewed how persistent pro-inflammatory cytokine and chemokine signaling (including IL-4, IL10, IL-13 and TGF-β) increases reactive oxygen and nitrogen species, enhancing programs related to oxidative stress and nitrosylation, which initially recruits tumoricidal macrophages (M1 phenotype) that switch to a pro-tumor (M2/TAMs) phenotype. This microenvironmental mechanism of immunosuppression interferes with proper recruitment of regulatory T-cells, through arginase upregulation (98). Karki and Kanneganti describe a similar dichotomous role of inflammasomes driving immunosuppressive programs by inhibiting the antitumor activity of T_helper_ cells (CD4+, CD8+), also involving myeloid-derived suppressor cells, natural killer cells and TAMs (89). Thus, increased levels of circulating inflammatory cytokines and recruitment of certain immune cell populations can propagate tumor phenotypes, resulting in poorer outcomes. Targeting specific cell populations or blocking the soluble factors that drive them is emerging as a promising approach for disrupting obesity-influenced cancer links (92, 93, 99).

### Nutritional Strategies That Mitigate Breast Cancer-Promoting Immune Mechanisms

Obesity is one of the most prevalent conditions by which low-grade inflammation becomes a chronic and systemic problem. Modulation of several specific dietary components represents a viable intervention strategy to offset the immunosuppressive and procancer effects of obesity (100). For example, many *in vivo* studies have demonstrated the capability of long chain *n*-3 polyunsaturated fatty acids (PUFAs) to reduce adipose-associated leptin and cytokine levels (i.e., TNFα, IL-6 and MCP-1), and increase anti-inflammatory adiponectin (101–104) (**Figure 1**). By reducing obesity-associated inflammation, PUFAs like eicosanoids exert chemoprotective effects, including, decreased proliferation and increased apoptosis, shown to reduce BC tumor burden and metastasis (105, 106). Kanaya and colleagues demonstrated the benefit of whole blueberry extract to modulate cytokine signalling and inhibit TNBC metastasis in mice (107). Consumption of high carbohydrate or high fat diets has been shown to increase breast cancer progression (108) in association with increased levels of specific cytokines, including IL-12 (109), osteopontin (110) or TIMPs (111, 112). Future studies should incorporate precision nutrition approaches to account for individual differences in metabolic, inflammatory and/or immune responses to dietary interventions. Sources of heterogeneity in response to dietary factors include genetic, epigenetic, and microbiome differences. For example, the D.I.E.T project focused on identifying optimized diets to augment immunotherapies, particularly through microbiome manipulation (113). Studies such as D.I.E.T. reinforce the need for precision nutrition efforts to address research gaps (114) and provide new opportunities to use food as medicine (115, 116) to break immune and inflammation-driven obesity-breast cancer links.

## Mediators of Vascular Integrity that Enhance Obesity-Breast Cancer Links

Tumor vasculature is an important component of the tumor microenvironment and is involved in various molecular processes (117). Tumor growth and proliferation require new blood vessels that can supply ample nutrients and oxygen and provide transport for metabolic waste. Moreover, metastasis requires tumor cells to infiltrate the vasculature to colonize distant sites (118, 119) (**Figure 1**). Angiogenesis, stimulated by TNFα, VEGF, and IL-8 (120), recruits new blood vessels during cancer initiation, progression and metastasis (121). New blood vessel formation is the first step in the metastatic cascade, and a critical mechanistic target triggered by inadequate oxygen supply (122). Hypoxia in the microenvironment stabilizes hypoxia-inducible factor (HIF)-1α, which regulates numerous metabolic, angiogenic, and apoptotic genes. HIF-1α enhances the expression of the chemokine receptor CXCR4 and interacts with the lipoxygenase pathway (122). The hypoxia that develops within a tumor promotes the malignant phenotype as the genomic stability of the growing tumor decreases (120, 122).

Intra-tumoral blood vessels display vessel dilation, high proliferation rate and increased permeability (118, 119, 122). Highly vascular tumors are associated with a greater number of macrophages (123). Obesity is associated with activation of the NLRC4 inflammasome, enrichment of TAMs, elevated IL-1β, and increased angiogenesis (124, 125). The high levels of IL-1β in response to obesity induce the expression of Angiopoietin-like 4 (ANGPTL4) from primary adipocytes in a manner dependent on NF-κB- and MAPK-activation, which is enhanced by hypoxia (125). Studies in mouse models have shown that adipocyte-derived ANGPTL4 drives BC progression under obese conditions and it could be a potential therapeutic target for treating obese BC patients (125). Microvessel density is a major prognostic factor for metastatic cancer, and a measure of angiogenesis (122, 123). For a tumor to gain metastatic potential, it must undergo an “angiogenic switch,” which occurs when factors enhancing angiogenic processes exceed the antiangiogenic factors of a tumor (126).

### Vasculature-Dependent Targets for Intervention

Dysfunctional tumor vasculature limits chemotherapy delivery to tumors. Additionally, a lack of sufficient oxygen delivery promotes hypoxia and acidification which ultimately leads to the development of more aggressive tumors. Physical exercise improves intratumoral vascularization and perfusion. Regular exercise is associated with lasting tumor vascular maturity, reduced vascular resistance, and increased vascular conductance. Thus, regular exercise is linked to reducing intratumoral hypoxia favoring the accessibility of circulating immune cells to the tumor microenvironment, inhibiting tumor development and improving cancer treatment (127).

Fatty acids, such as arachidonic acid and omega-3 fatty acids, have been found to have a role in breast cancer (128, 129). An increased expression of arachidonic acid in breast cancer tissues is strongly correlated with an enhanced mTORC1 and mTORC2 signaling (128). Furthermore, arachidonic acid-activated mTOR plays a primary role in angiogenesis and tumorigenesis (128). The expression of VEGF and cytosolic phospholipase A2 (cPLA2) are also increased by arachidonic acid (128). Omega-3 fatty acids have been linked to protective roles in breast cancer progression, such as the inhibition of angiogenesis and metastasis (**Figure 1**). Additionally, several studies have suggested an association between a higher omega-3 intake and a lower risk of breast cancer (129, 130). Supplementation of omega-3 fatty acids in women undergoing surgery for locally advanced, invasive carcinoma resulted in decreased expression of both Ki-67 and VEGF compared to a control group (130). Furthermore, the omega-3 fatty acid supplementation group displayed a longer disease-free survival and overall survival (130).

Oxidative stress occurs due to an imbalance between antioxidant defenses in the body and production of reactive species, such as reactive oxygen species (ROS) or reaction nitrogen species (RNS) (131). This stress has been suggested as an important factor in tumor initiation and progression. Nitric oxide synthases (NOS), such as endothelial NOS, can generate RNS, and have been shown to modulate processes including inflammation, angiogenesis and metastasis (131). Human breast cancer cells displayed activation of the EGFR and ERK pathways when treated with nitric oxide, and ultimately showed an increase in invasive potential (132). In one study using rats with mammary tumors, a diet enriched with PUFAs resulted in tumor regression due to improved drug delivery. The observed tumor regression was associated with decreased activation of endothelial NOS, which normalized the vasculature of the mammary tumors (133).

## Obesity Links to Breast Cancer Metastasis and Interventions to Disrupt the Links

Metastasis is linked to 90% of tumor-related deaths in BC patients (134), and ~ 30% of patients develop metastases at some point after diagnosis (135). Obese patients have larger primary tumors at diagnosis and increased risk of lymph node metastasis for all BC subtypes (136). Obesity decreases the time from primary diagnosis to metastatic disease, and mouse models have confirmed that lung metastases are more prevalent in obese mice compared with lean mice (137, 138). Given that increased metastatic potential is associated with obesity, uncovering the mechanisms of this relationship is now a major focus of research.

Adipose stem cells (ASCs), abundant in adipose stromal tissue, can become osteoblasts, chondrocytes, myocytes, or monocytes, and are a new player in obesogenic metastasis (134, 139), specifically in TNBC. ASCs from obese mice increase tumor microenvironment leptin levels, directly promoting metastasis rather than enhancing primary tumor growth (134). Chronic-low-grade, obesity-associated inflammation activates immune cells preparing the metastatic niche (140); which then limits immunosurveillance protections, suppressing CD8+ T-cell function through IL-1β, while promoting neutrophil expansion and polarization (141). Neutrophil-mediated mechanisms in lungs of obese mice were specifically shown to result in higher metastatic burden (138, 142).

Epithelial-to-mesenchymal transition (EMT) is another mechanism associated with a more invasive and aggressive metastatic phenotype. EMT involves loss of epithelial polarity, de-differentiation, and local invasion (134, 143), but its mechanistic underpinnings have only recently become linked with obesity. For example, Bousquenaud and colleagues demonstrated tumors from mice fed a high-fat diet lost the cell junction protein E-cadherin, but increased expression of mesenchymal markers N-cadherin and vimentin (138). Obesity-driven inflammatory markers (i.e., NF-κB, STAT3, and COX-2) also play a role in EMT (3, 144). These emerging mechanisms have begun to reveal the complex relationships between obesity and the metabolic reprogramming of tumor cells that favor metastatic progression. Future interventions strategies will also need to account for the multi-factored contributors, namely inflammation, when addressing this aspect of BC.

## Discussion on Challenges and Gaps for Nutritionally Relevant Interventions

The aggressive biology of the tumor microenvironment metabolically activated by dysregulated adipose tissues in the obese state reduces the efficacy of cancer treatments, posing greater challenges in patient care and disease management. Further investigations are needed to improve early diagnosis and treatment mechanisms to successfully target BC within these patients. Nutrition and physical activity-based interventions that better manage obesity represent viable strategies to break obesity-breast cancer links.

The most recent guidance for adult (19-65 years) daily nutritional intakes includes protein at 10-35%, fat at 20-35% and total carbohydrates at 45-65% (145–147). These ranges demonstrate the complexity involved in deciphering what determines a healthy diet/dietary pattern. Dietary Reference Intake (DRI) guidelines classify diet and the role of nutrition in the context of chronic diseases more specifically (148–153), but navigating these extensive reports is a major challenge. Current recommendations focus on making nutritional choices that reflect overall healthy eating patterns to reduce chronic disease incidence (154), including limiting refined carbohydrates/sugars to 25% of total daily intake, and levels of cholesterol, trans- and saturated fatty acids to, “as low as possible while consuming a nutritionally adequate diet” (147, 155).

A substantial number of preclinical (47, 96, 156–163) and epidemiological (164–170) studies have shown the impact of diets, that result in obesity, with different macronutrient compositions on BC development and disease outcomes. For example, several studies have demonstrated the negative impacts on overall health from diets high in fat content, including cancer outcomes from consumption of *Western*-style diets, high in poor quality carbohydrates (refined and simple sugars) and saturated fats (171–176). Comparatively, there is still controversy regarding high protein (thus, lower carbs and fat) diets. Animal and human studies have demonstrated the benefits of high protein content (at intakes of 23-69%) on slowing or inhibiting mammary tumor formation, reducing disease progression, improving chemosensitivity, and extending survival/lifespan (177–179). In contrast, Park et al. correlated higher acid load, presumably from high protein intake and concomitant high phosphorus consumption, with higher ER- BC risk (180) and other lifetime cancer risks (181, 182) in the Sister Study. The *Mediterranean*-style diet involves consumption of high levels of protein and fat, but the sources are restricted to lean, non-processed (low red meat) proteins and ‘healthier’ forms of fat, with increased fiber content from fruits, vegetables and whole grains. This dietary pattern has been associated with lower BC risk, specifically through microbiome population modifications (i.e., *Lactobacillus*) that diminish cancer-promoting mechanisms, like oxidative stress, in the mammary gland (174, 183). However, there are still significant gaps at both the population and individual level concerning dietary guidelines to maintain health. Several reviews and investigations have focused on defining appropriate nutrient intakes for improved BC patient outcomes (100, 166, 184–186). Yet, changes in life-style and food choices represent a challenge for BC patients; for example, Shi et al. found that newly diagnosed with BC consistently consume excessive fat and slightly increased consumption of fruits and vegetables following diagnosis (166). These and other findings stress the need of incorporating nutritional and psychosocial counseling to better manage diverse ramifications of diagnosis and treatment of BC patient to increase recovery rate and overall health.

## Conclusions

In conclusion, there has been tremendous progress in understanding the mechanisms underlying the obesity-BC link. While there is more to learn about the biology of this link, emphasis should be placed on translating our knowledge into effective strategies to reduce the obesity-associated burden of BC in women. Emerging initiatives in precision nutrition focused on understanding why metabolism and nutrition requirements differ between individuals —considering host factors (i.e., genetic, epigenetic, microbiome) and environmental factors (i.e., diet, physical activity, mental health, and direct environmental exposures)— will enable more personalized, targeted guidance for optimal mechanism-based nutritional strategies to reduce obesity-driven BC (114).

## Author Contributions

DS and XMBM outlined the topics discussed in this review. JM, MC, and ED contributed to writing specific sections. SH, XMBM, and DS provided critical revision of the article. XMBM designed the art work for **Figure 1**. All authors contributed to the article and approved the submitted version.

## Funding

XMBM is supported by the Marilyn Gentry Fellowship Program in Nutrition and Cancer from the American Institute for Cancer Research–World Cancer Research Fund (AICR/WCRF) and the University of North Carolina. DS is supported by the Nutrition Research Institute Faculty Development Program. SH is supported by grants from the National Cancer Institute (NCI-R35CA197627) and from the Breast Cancer Research Foundation (BCRF#18073).

## Conflict of Interest

The authors declare that the research was conducted in the absence of any commercial or financial relationships that could be construed as a potential conflict of interest.
